# A Durable Magnetic Superhydrophobic Melamine Sponge: For Solving Complex Marine Oil Spills

**DOI:** 10.3390/nano12142488

**Published:** 2022-07-20

**Authors:** Hanmo Si, Qingwang Liu, Zhenzhong Fan, Biao Wang, Qilei Tong, Mengqi Lin

**Affiliations:** Key Laboratory of Enhanced Oil Recovery of Education Ministry, Northeast Petroleum University, Daqing 163318, China; sihanmoy@163.com (H.S.); fanzhenzhong@nepu.edu.cn (Z.F.); lovepeacht@163.com (B.W.); neputongqilei@163.com (Q.T.); lmqixxn666@163.com (M.L.)

**Keywords:** superyhydrophobicity, melamine sponge, polydopamine, nano-particles, oil–water separation

## Abstract

The problem of offshore oil leakage has wreaked havoc on the environment and people’s health. A simple and environmentally friendly impregnation method combined with marine mussel bionics was used to address this issue. Using the viscosity of polydopamine (PDA), nano- Fe_3_O_4_ and WS_2_ adhered to the framework of the melamine sponge (MS), and then the magnetic sponge was modified with n-octadecanethiol (OTD), and finally the superhydrophobic magnetic melamine sponge (mMS) was prepared. The modified sponge has superhydrophobicity (WCA, 156.8° ± 1.18°), high adsorbability (40~100 g°g^−1^), recyclability (oil adsorbability remains essentially unchanged after 25 cycles), efficient oil–water separation performance (>98%), and can quickly separate oil on the water’s surface and underwater. Furthermore, the modified sponge exhibits excellent stability and durability under harsh operating conditions such as strong sunlight, strong acid, strong alkali, and high salt, and can control the direction of the sponge’s movement by loading a magnetic field. To summarize, mMS has many potential applications as a new magnetic adsorption material for dealing with complex offshore oil spill events.

## 1. Introduction

With the rapid development of industry, the amount of oil extracted and used is increasing yearly. At the same time, oil leakage accidents frequently occur in offshore oil fields. Oil leakage into seawater destroys the marine ecological environment, causing water pollution and marine life death, and poses a threat to human health due to toxic substances such as benzene, aromatic hydrocarbons, and hydrogen sulfide contained in crude oil [1,2,3,4]. There are many methods to solve offshore oil spills, of which adsorption [5] is one of the most commonly used solutions. The commonly used adsorbents [6,7,8] have the characteristics of high porosity and high specific surface area [9], but traditional adsorbent materials, such as inorganic minerals [10] and natural polymer materials [11], have disadvantages such as low adsorption efficiency, poor oil–water selectivity, and high cost [12,13,14]. As a result, finding a suitable adsorbent material to handle the problem of offshore oil spills is critical.

In recent years, commercial polymer sponges, such as melamine and polyurethane sponges, have been paid attention to as good oil-absorbing materials due to their excellent elasticity, large porosity, low cost and active groups on the surface of sponges for easy modification. Oil adsorption efficiency is significantly reduced due to the sponge’s amphiphilic property. By coating nano-structured materials [15] (nano-SiO_2_, rGO, metal nanoparticles, etc.), chemical etching [16] and grafting modification of low surface energy materials [17] can change hydrophilic and oil-philic sponges into superhydrophobic and superoil-philic sponges, improving the oil adsorption efficiency and oil–water selectivity of sponges. Ruan et al. [18] reported a two-step method for the synthesis of superhydrophobic melamine sponges, in which the sponges were first immersed in a dopamine hydrochloride solution to deposit a polydopamine film on the surface. Then, the sponge surfaces were modified with 1H, 2H, 3H, and 4H perfluorooctanethiol, resulting in superhydrophobic sponges with excellent adsorption properties, recoverable properties, and oil–water selectivity. However, the fluorinated thiols used in this method have disadvantages, such as high cost and environmental pollution. Chen et al. [19] reported a graphene oxide modified polyurethane sponge by grafting a low surface energy substance, perfluorooctyltriethoxysilane, on the sponge surface, and the water contact angle of the modified sponge is greater than 150°, which has superhydrophobic properties. Yet, graphene is expensive and is currently challenging to use on a large scale. A good oil–water separation material should not only have good performance, but should also consider the production cost, process, and environmental protection [20].

There have been numerous reports on the development of magnetic superhydrophobic oil-absorbing materials. Liu et al. [21] used a straightforward one-step soaking method. By soaking a polyurethane sponge in a suspension of the low surface energy compound Actyflon-G502 (Dodecafluo Oroheptyl-Propyl-trimethoxylsilane), the sponge is easily controlled by a magnet and can effectively separate various oil–water mixtures. Yin [22] and colleagues prepared a superhydrophobic magnetic melamine sponge with in situ synthesis ferroferric oxide particles and small candle wax dip coating. The sponge wetting Angle has a high wetting Angle (158.8°), excellent oil absorbency and circulating sex, and it is important to note that the method put forward vegetable wax as the wettability of materials by changing the coating reagent. It is a novel method for producing superhydrophobic sponges. Li et al. [23] used a solvothermal reaction in the presence of surface modifiers to successfully synthesize hydrophobic magnetic nanoparticles, which then adhered to sponges with silicone adhesive. This method can create hydrophobic structures on the surface of the melamine sponge without modifying the surface. However, when used to deal with the problem of sea oil overflow, it is necessary to consider the harsh marine environment, such as acid and alkali, temperature, intense sunlight, and other problems, which will affect the stability of oil-absorbing materials, reducing the oil–water separation ability and oil-absorbing efficiency of oil-absorbing materials. To address this issue, high-temperature and high-pressure resistant nano-WS_2_ and nano-Fe_3_O_4_ nanoparticles are chosen to adhere to the sponge skeleton, improving the sponge’s stability and durability and endowing it with magnetism.

Thus, this paper proposes a simple and environmentally friendly method to prepare superhydrophobic magnetic melamine sponges by a two-step process successfully. Fe_3_O_4_ and WS_2_ nanoparticles were dispersed in the first step on the sponge skeleton. The addition of nanoparticles made the sponge surface form a rough micro-nano hierarchical structure.The second step is to construct PDA/OTD film on the sponge surface, reducing the sponge’s surface energy. Building rough structures and reducing surface energy are the two main ways to modify hydrophobic materials. The modified sponge (mMS) has excellent hydrophobic, adsorption, recycling, oil–water separation properties and chemical stability, which can provide a good reference value for treating offshore oil spill events.

## 2. Experimental Section

### 2.1. Reagents and Instruments

Melamine Sponge (MS), Shangpin Tiancheng Trading Co., Ltd. (Guangzhou, China). Dopamine hydrochloride, Shanghai Maclean Biochemical Technology Co., Ltd. (Shanghai, China); Trihydroxymethylaminomethane (Tris), Voredas experimental reagents consumables; N-octadecyl mercaptan, Runyou Chemical Co., Ltd. (Shenzhen, China). Nano-Fe_3_O_4_ (20 nm 99.0%), Aladdin Reagent Co., Ltd. (Shanghai, China); Nano-WS_2_ (10 nm 99.0%), anhydrous ethanol, Tianjin Kaitong Chemical Reagent Co., Ltd. (Tianjin, China); Methyl orange, oil red, cyclohexane, Tianjin Kemiou Chemical Reagent Co., Ltd. (Tianjin, China); Methylene blue, Tianjin Zhiyuan Chemical Reagent Co., Ltd. (Tianjin, China); Carbon tetrachloride, Aoran Fine Chemical Research Institute, (Tianjin, China); Kerosene and Diesel oil, China Petrochemical Corporation (Beijing, China); Corn germ oil, Xiwang Food; Paraffin oil, Jinan Luying Chemical Industry (Jinan, China); Crude oil, Daqing oil field oil–water separation combined station (Daqing, China); NaCl, MgCl_2_, Tianjin Kaitong Chemical Reagent Co., Ltd. (Tianjin, China).

Jsm-7800f Prime Field emission Scanning electron Microscope, Japan Electronics Co., Ltd. (Tokyo, Japan); Ie250x-max50 Oxford Spectrometer, Oxford Instrument Technology Co., Ltd. (Shanghai, China); Thermo Nicolet iS10 Fourier Transform Infrared Spectrometer, Thermo Fisher Technologies (Waltham, MA, USA); Jy-phb contact Angle tester, Chengde Youte Testing Instrument Manufacturing Co., Ltd. (Chengde, China); Ultrasonic cleaning machine, Desen Seiko Co., Ltd. (Fuzhou, China); DF101S Thermal magnetic stirrer, Laibo Scientific Instrument Co., Ltd. (Shanghai, China); 2HG101A-0 Electric heating Constant Temperature Drying oven, Changzhou Jintan Liangyou Instrument Co., Ltd. (Changzhou, China).

### 2.2. Preparation of mMS

**Figure 1:**
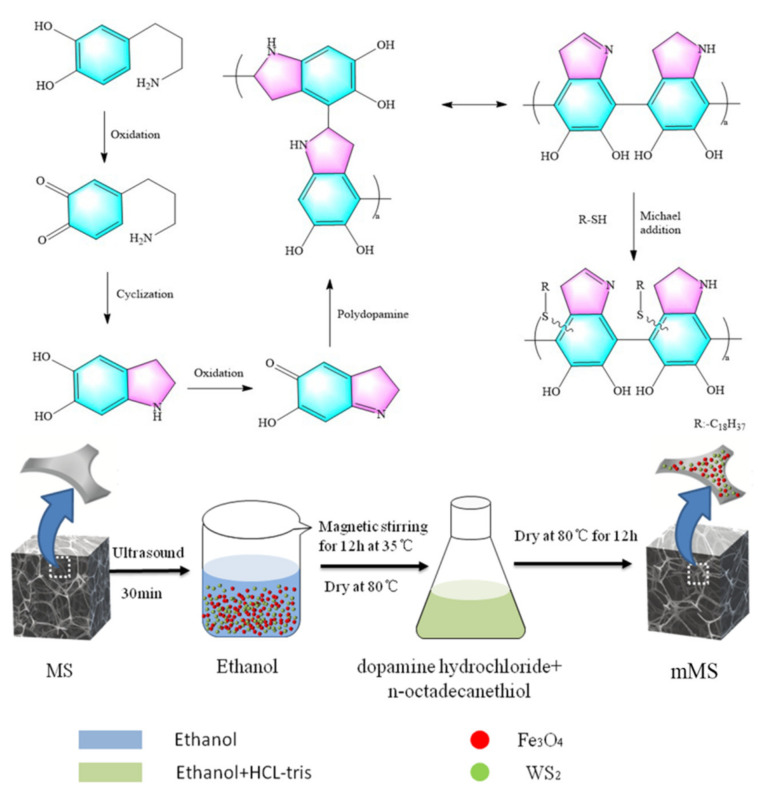
Schematic diagram of preparation of mMS.

The melamine sponges (MS) were cut into pieces of 1.0 cm× 1.0 cm× 1.5 cm in size and then ultrasonically cleaned in ethanol and distilled water for 1 h. After being dried in an oven at 60 °C, the MS was immersed in an ethanol solution of nano-Fe_3_O_4_ (100 mg) and nano-WS_2_ (40 mg) by ultrasonication for 30 min and then removed. After drying, the MS/Fe_3_O_4_ were immersed in a mixture of Trimethylol aminomethane hydrochloride (Tris-hcl) and ethanol containing dopamine hydrochloride (2 mg/mL) and n-octadecanethiol (0.5 mg/mL), heated in a water bath at 35 °C and removed by magnetic stirring for 12 h. The mixture of Tris-hcl and ethanol is 1:1. Subsequently, the sponges were washed with distilled water and dried in a drying oven at 60 °C to obtain mMS. The reaction preparation process and mechanism of mMS are shown in Figure 1.

### 2.3. Contact Angle Test

4 μL of water was dropped onto the mMS’s surface. The images of the water droplets were recorded after they had stabilized, and the water contact Angle of the sponge was calculated using the tangent method. To ensure measurement accuracy, the contact Angle of the sponge at different positions on the upper and lower surfaces was measured fivetimes.

### 2.4. Oil Adsorption and Recycling Performance Test

The mMS was immersed in different organic solvents and oils. The sponge’s adsorption multiplier for various organic solvents and oils is calculated after it has been saturated with oil. The formula for calculating the adsorption multiplier is:

(1) $$W_{1}=\frac{m_{1}-m_{0}}{m_{0}}\times 100\%$$

To verify the recyclability of the mMS, kerosene, diesel oil, corn germ oil, paraffin oil and n-hexane were selected as the test oil and organic solvent. The oil was recovered using the adsorption-pressure method to calculate the mMS recycling efficiency after 1, 5, 10, 15, 20, and 25 cycles of adsorption-pressure.

In Formula (1), $W_{1}$(g°g^−1^) is sponge adsorption ratio, $m_{0}$(g) and $m_{1}$(g) are sponge weight before and after adsorption, respectively.

### 2.5. Oil–Water Separation Performance Test

To investigate the oil–water separation performance of mMS in a dynamic oil–water mixing system, diesel oil, kerosene, corn germ oil and paraffin oil were selected as the oil phases. Weigh 2 g of different oils and organic solvents, respectively;add 50 mL of deionized water, and stir with a magnetic stirrer to maintain a dynamic oil–water mixing state. The formula for calculating the oil–water separation efficiency is:

(2) $$E=\frac{C_{1}-C}{C_{2}}\times 100\%$$

where *E* (%) is the separation efficiency, *C*(g) is the total mass of oil and water mixture, *C*_1_(g) is the total mass of oil and water mixture after separation, and *C*_2_(g) is the mass of oil before separation (the mass of mMS remains unchanged after soaking in aqueous solution).

To observe the oil–water separation effect of mMS more directly, a simple device was designed to separate water-CCl_4_(3:2) mixed solution. The conical funnel was fixed on the iron frame, mMs were used as the filter layer, and the water-CCl_4_ mixed solution was poured into the funnel. After that, the oil–water separation efficiency was calculated by Formula (2).

### 2.6. Stability and Durability Test

The effects of light, pH, temperature, and sodium chloride concentration on the stability and durability of mMS were examined. The wetting Angle of the modified sponge was measured every 2 h while it was exposed to direct sunlight for 10 h. The modified sponges were soaked in acidic and alkaline solutions with pH of 1, 3, 5, 7, 9, 11, and 13, respectively, using Hcl and NaOH. After it had dried, the contact Angle was calculated using a contact Angle tester. To mimic the stability of modified sponges in seawater, solutions with NaCl concentrations of 10%, 20%, 30%, and 40% were generated. After immersing the sponges in the solution for 24 h, the water contact Angles were measured. The water contact Angles were measured after soaking the modified sponges in water baths at 50 °C, 60 °C, 70 °C, 80 °C, and 90 °C for 24 h.

### 2.7. Magnetic Test

Use a Petri dish to hold the water/kerosene mixture system, place the mMS on the water surface, and use a magnet to control the direction of sponge movement.

## 3. Results and Discussion

### 3.1. Chemical Characterization Analysis

#### 3.1.1. Scanning Electron Microscope (SEM) Analysis

**Figure 2:**
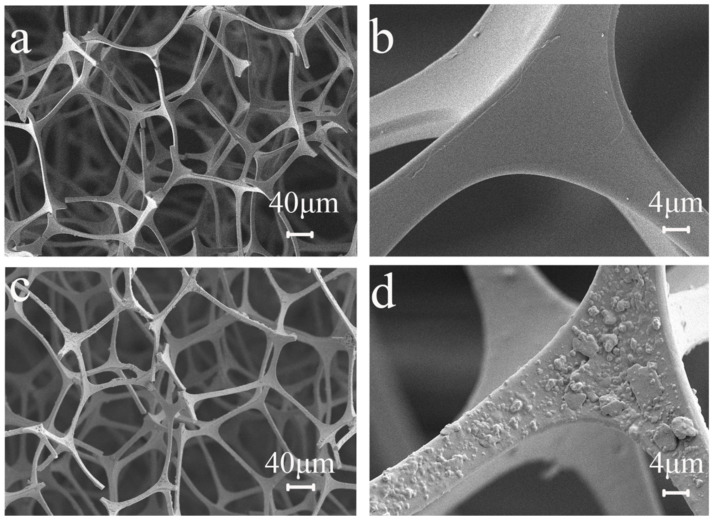
SEM images of MS (**a**,**b**) and mMS (**c**,**d**).

The surface morphology of sponges before and after modification was observed by scanning electron microscopy (SEM) at different magnifications. Figure 2 shows SEM images of MS and mMS. MS’s skeleton surface is smooth and flat (Figure 2a,b), with a porous structure that allows rapid oil adsorption. The sponge skeleton becomes rough after modification (Figure 2c,d). Because of the action of polydopamine, nano-Fe_3_O_4_ and nano-WS_2_ adhere to the sponge skeleton, forming a rough micro nano-layered structure. The micro nano-layered structure’s convex surface can effectively prevent disturbances, prevent droplet entry, and maintain the stability of the superhydrophobic surface [24]. Sponge hydrophobicity is increased by this structure.

#### 3.1.2. EDS Analysis

**Figure 3:**
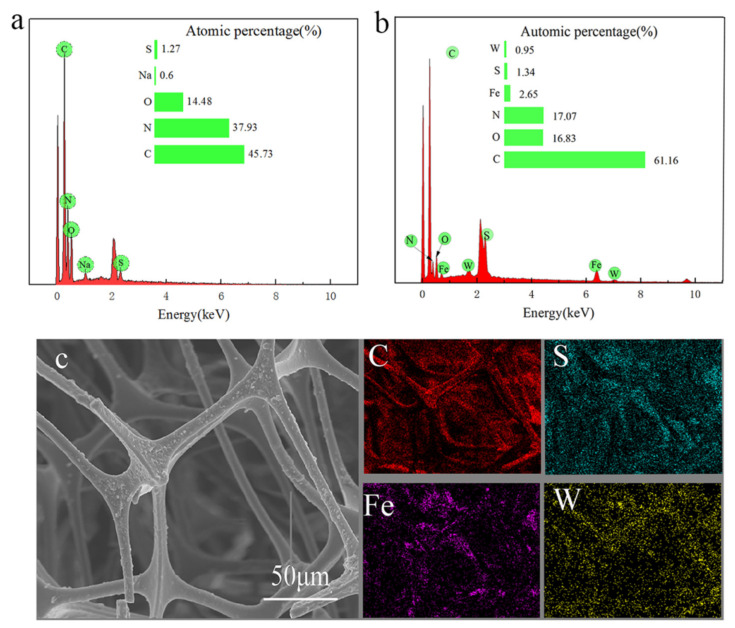
EDS diagram of MS (**a**) and mMS (**b**); (**c**) EDS elemental mapping of mMS in the selected area.

EDS spectra before and after modification of MS are shown in Figure 3. Before modification, the surface of the sponge contains a large amount of C, H, O, S, and other elements (Figure 3a); after modification, Fe and W elements appear on the surface of the sponge (Figure 3b), confirming that the modified sponge is loaded with nano-Fe_3_O_4_ and nano-WS_2_ particles. The decrease in N element content was caused by the uniform coverage of polydopamine on the sponge’s surface, which covered the N content of the sponge itself. The C element of the sponge increased significantly before and after modification, and the atomic percentage increased from 45.73% to 61.16%, indicating that n-octadecanethiol was successfully introduced to the surface of the sponge. MS’s hydrophobic modification was achieved. Figure 3c shows mMS’s element mapping diagram. The elements C, S, Fe, and W, are evenly distributed on the sponge’s skeleton, revealing that a uniform hydrophobic coating forms on the sponge’s surface.

#### 3.1.3. FTIR Analysis

**Figure 4:**
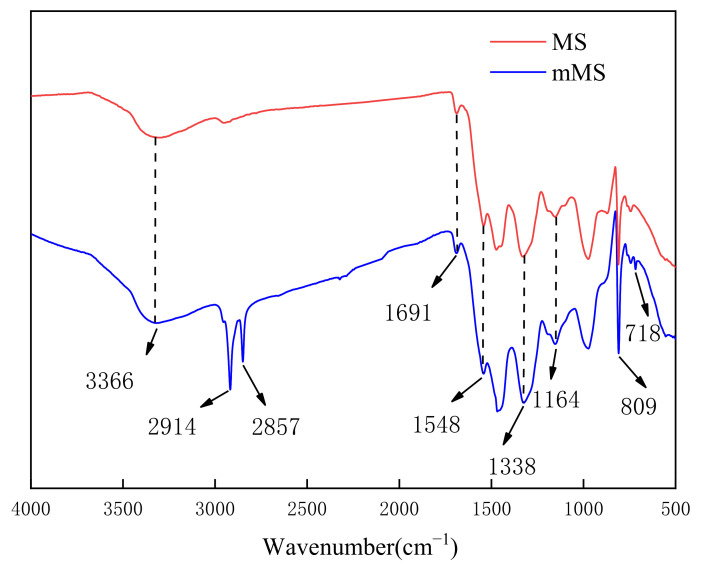
FTIR diagram of MS and mMS.

The FTIR plots of MS and mMS are shown in Figure 4. The sponges before and after modification all have the absorption peaks of MS containing characteristics, which are as follows: Triazine ring characteristic vibration peak at 809 cm^−1^, C-O stretching vibration peak at 1164 cm^−1^, -CH_2_-bending vibration peak at 1338 cm^−1^, C-N stretching vibration peak at 1548 cm^−1^, C=N characteristic absorption peak at 1691 cm^−1^ and N-H stretching vibration peak at 3366 cm^−1^ [25,26,27]. The two more absorption peaks than MS at 2914 cm^−1^ and 2857 cm^−1^ are the asymmetric and symmetric stretching vibration peaks of -CH_2_ on n-octadecylmercaptan [28], indicating that the surface of mMS is grafting with a long carbon chain of n-octadecylmercaptan. At 718 cm^−1^, the S-C-H vibration absorption peak was a new absorption peak. Because the aromatic group of PDA is linked to the sulfur group of n-dodecyl mercaptan [29], the Michael addition reaction between them forms a PDA/OTD coating on the surface of mMS, converting the original MS from hydrophilic to superhydrophobic.

### 3.2. Wettability Analysis

**Figure 5:**
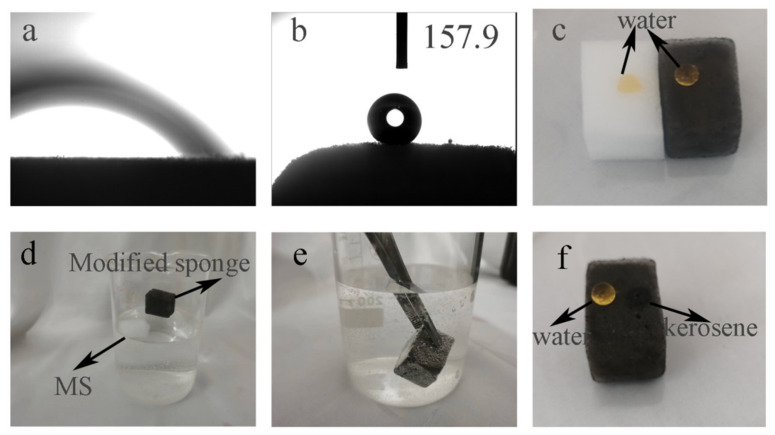
Images of water contact angle of MS (**a**) and mMS (**b**); water droplets of methyl orange stain on the surface of MS and mMS (**c**);MS and mMS immersed in water (**d**); silver mirror phenomenon formed by pressing mMS into water under external force (**e**); water and kerosene drops on the surface of mMS (**f**).

Wettability is one of the essential characteristics of a solid surface. In general, the wettability of the object surface is measured by measuring the contact Angle of liquid on a solid surface. The water contact angle of the object surface greater than 150° is called a superhydrophobic surface [30,31,32,33]. As shown in Figure 5a,b, the water contact Angle of the original MS is 0°, while that of mMS is 157.9°. The increase of the water contact Angle indicates that the surface wettability of the MS changes from a hydrophilic surface to a superhydrophobic surface. Water droplets (dyed with methyl orange) were dropped on the surface of the mMS and the original MS, respectively (Figure 5c). We observed that water droplets were adsorbed by the original MS, while the surface of the mMS was spherical, showing that the sponge changed from the original hydrophilic material to hydrophobic material before and after modification. After soaking the original MS and mMS in water (Figure 5d), it was clear that the original MS exhibited hydrophilic water adsorption and sunk into the water, whereas mMS was light in weight and hydrophobic, allowing it to float on the water surface. A silver mirror can be observed when mMS is pressed under water with tweezers (Figure 5e). The phenomenon occurs because mMS’s surface is hydrophobic, causing a large amount of air to be trapped between the surface and the surrounding water, creating a light reflection phenomenon [34]. When water and kerosene were dropped on the surface of mMS (Figure 5f), it was observed that water formed a spherical water droplet on the surface of the sponge and was not adsorbed by the sponge, while kerosene quickly penetrated the sponge, illustrating that the modified sponge has super hydrophobic/super lipophilic properties.

### 3.3. Adsorption Performance Analysis and Recycling Performance Analysis

**Figure 6:**
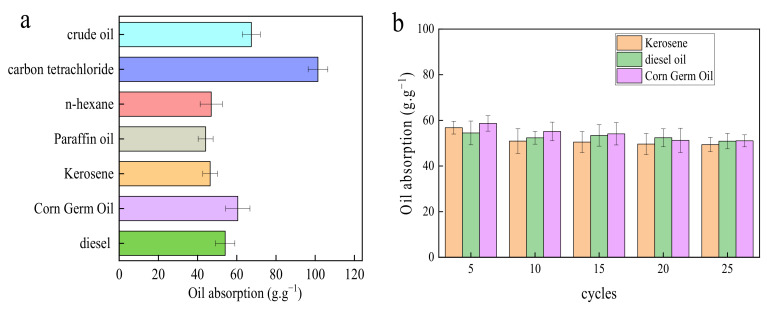
Adsorption ratio of mMS to different oils and organic solvents (**a**); adsorption ratio of mMS to kerosene, diesel and corn germ oil after multiple pressing cycles (**b**).

The saturated adsorption capacity of modified sponge for crude oil, kerosene, diesel oil, corn germ oil, paraffin oil, n-hexane, and carbon tetrachloride is 40~100 g°g^−1,^ as seen in Figure 6a. Because the density of carbon tetrachloride is much higher than that of other oil products, the adsorption rate of carbon tetrachloride by mMS is significantly higher. Adsorption pressing is used to recover the sponge. As shown in Figure 6b, after 25 cycles, the modified sponge still has good elasticity, and the adsorption ratio of kerosene, diesel oil and corn germ oil decreases slightly, but it can still keep more than 40 g°g^−1^. MS is a kind of porous material. When the sponge comes into contact with oil, its pores adsorb it via capillary force. This adsorption pressing behavior will destroy the sponge’s pore structure and reduce the adsorption ratio. The results show that mMS has good adsorption for different oils and organic solvents and is also recyclable. In practice, after the sponge is recovered by the recovery device, the oil adsorbed by the sponge can be collected by mechanical pressing, achieving the goal of recycling while effectively reducing costs and avoiding secondary pollution caused by treating the oil or organic matter adsorbed in the material by combustion or evaporation.

### 3.4. Oil–Water Separation Performance Analysis

**Figure 7:**
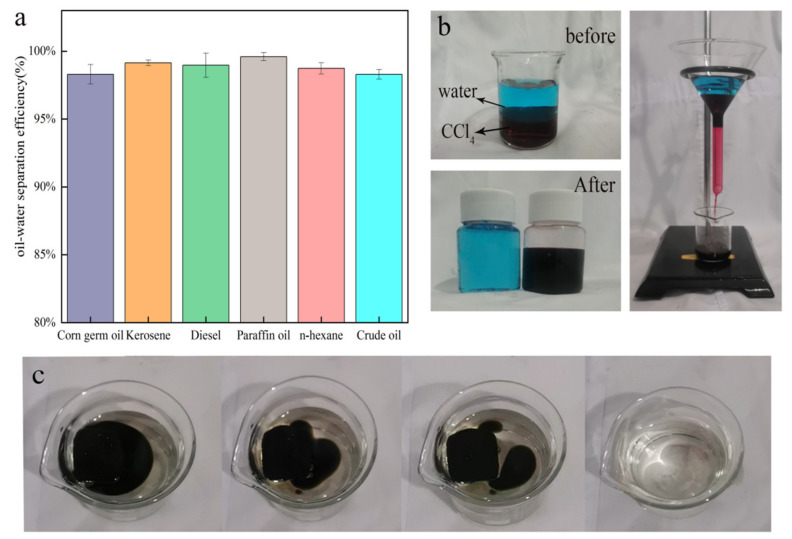
Oil–water separation efficiency of mMS in dynamic oil–water mixtures (**a**); a simple device separates water-CCl_4_ mixture (**b**); adsorption of crude oil on simulated seawater surface by mMS (**c**).

MS is a novel foam material with a high opening rate and a three-dimensional network structure. It is amphiphilic, which means it can adsorb both oil and water. This property significantly reduces the sponge’s oil adsorption rate. Making the sponge super hydrophobic and lipophilic can improve the material’s oil adsorption rate in the oil–water mixed solution. As a result, the oil–water separation efficiency is an important indicator for testing the adsorption material’s performance. The modified sponge can effectively separate the oil–water mixture, as shown in Figure 7a, and the oil–water separation efficiency of corn germ oil, kerosene, diesel, paraffin, n-hexane, and crude oil is greater than 98%. Figure 7b shows the use of a simple device to separate the mixed solution of water and CCl_4_. Due to the wettability of the mMS, water cannot pass through the sponge. Under the action of gravity, carbon tetrachloride is separated from the oil–water mixture, and the separation efficiency can reach 98.3%. The modified sponge can also be used as a filter layer by a device to separate oil and water. As shown in Figure 7c, to better simulate sponges’ use in oil leakage events, simulated seawater (NaCl, 26.518 g/mL; MgCl_2_, 2.44 g/L) is prepared with the formula of the third Oceanic Administration of the Chinese Academy of Sciences. It can be observed that the crude oil on the surface of simulated seawater is adsorbed by mMS, which shows that mMS can effectively recover the leaked crude oil in seawater.

### 3.5. Stability and Durability Analysis

**Figure 8:**
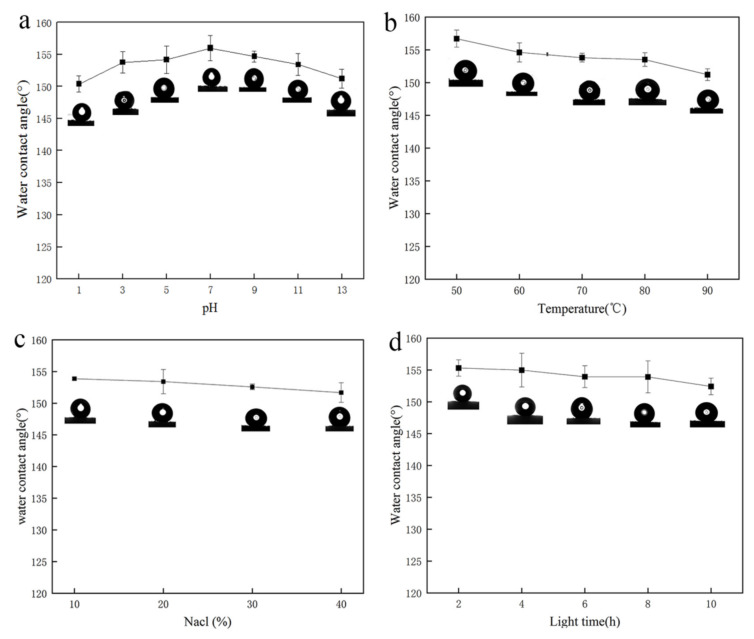
The influence of pH (**a**), temperature (**b**), NaCl concentration (**c**), and strong light (**d**) on the water contact Angle of mMS.

Figure 8a depicts the change in water contact Angle after soaking the modified sponge in aqueous solutions with pH = 1, 3, 5, 7, 9, 11,and 13 for 24 h. The modified sponge’s water contact Angle decreases due to the influence of acid and alkali, but it retains superhydrophobicity. The water contact Angle did not change significantly after the modified sponge was irradiated with intense light for 10 h, as shown in Figure 8b. In Figure 8c, high-temperature soaking significantly reduces the water contact angle of the modified sponge after 24 h of soaking in aqueous solutions at different temperatures. However, the modified sponge can still perform well in high temperatures because of the flame retardancy of MS and the high-temperature resistance of nano-WS_2_. The modified sponges also remained stable in sodium chloride-simulated seawater. From Figure 8d, the hydrophobicity of the modified sponge decreases slightly in a high salinity environment, but its water contact Angle remains above 150°. Here, we can see that mMS has exceptional stability and durability in the face of complicated marine environments. This has vital practical significance for the future separation of crude oil from water in severe environments.

### 3.6. Magnetic Performance Analysis

**Figure 9:**
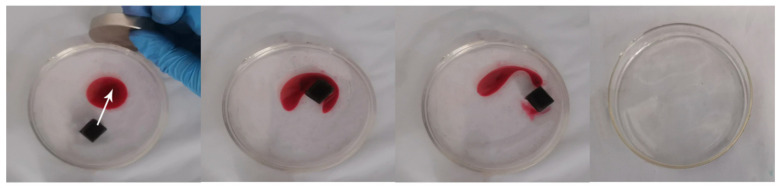
Use a magnet to control the moving direction of mMS to absorb kerosene on the water surface.

As shown in Figure 9, after the mMS is placed on the water surface, place the magnet on the outside of the Petri dish to control the moving direction of the sponge. Under the influence of magnetism, the sponge moves to the side containing kerosene. When mMS contacts kerosene on the water surface, kerosene on the water surface is rapidly adsorbed within 15 s. This magnetic effect enables sponges to be driven by magnets in practical applications, changing the direction of movement of sponges to absorb oil in different containing areas, thereby improving sponges’ practicality and adsorption efficiency.

## 4. Conclusions

In conclusion, a superhydrophobic magnetic sponge material was successfully prepared by a simple method. SEM images confirmed the high porosity of mMS and the increased roughness of the sponge before and after the modification. EDS images confirmed that the surface of the sponge had been successfully modified with nano-Fe_3_O_4_, nano-WS_2_, and n-octadecanthiol. The Michael addition reaction between n-octadecanethiol and polydopamine was confirmed by FIFR, resulting in a PDA/OTD coating on the sponge’s surface. The wettability of mMS was analyzed, and the water contact angle of the sponge was greater than 150°, with superhydrophobic and superoleophilic properties. The adsorption and cycling experiments showed that mMS has a high adsorption performance on various organic solvents and oils. The structure of mMS is still stable after 25 cycles, as is the adsorption ratio of various oils and organic solvents can still be maintained at more than 40 g°g^−1^. Experiments with oil–water separation showed that mMS could maintain excellent oil–water selectivity at both the surface and underwater, with an oil–water separation efficiency of more than 98% for various oils in dynamic oil–water mixtures. mMS has been shown to perform well in harsh environments in stability and durability tests. Superhydrophobicity can be maintained in the presence of intense sunlight, a hot water bath, acid, alkali, and a high salt solution. Furthermore, due to the magnetic properties of mMS, it can be used to drive sponges through magnetism. It is expected to be widely used in oil field leakage events in the future.

## Data Availability

The raw/processed data required to reproduce these findings cannot be shared at this time as the data also forms part of an ongoing study.
